# Ectopic virilising adrenocortical tumour in the spinal region in an 8 year-old boy: a case report and review of the literature

**DOI:** 10.1186/s13052-015-0169-8

**Published:** 2015-09-02

**Authors:** Agata Skórka, Elżbieta Moszczyńska, Karolina Kot, Marcin Roszkowski, Elżbieta Jurkiewicz, Wiesława Grajkowska, Maciej Pronicki, Olgierd Pilecki, Mieczysław Szalecki

**Affiliations:** Department of Paediatrics, Medical University of Warsaw, Dzialdowska 1, 01-189 Warsaw, Poland; Department of Endocrinology and Diabetology, The Children’s Memorial Health Institute, Al. Dzieci Polskich 20 04-730, Warsaw, Poland; Department of Neurosurgery, The Children’s Memorial Health Institute, Al. Dzieci Polskich 20 04-730, Warsaw, Poland; Department of Diagnostic Imaging, The Children’s Memorial Health Institute, Al. Dzieci Polskich 20 04-730, Warsaw, Poland; Department of Pathology, The Children’s Memorial Health Institute, Al. Dzieci Polskich 20 04-730, Warsaw, Poland; Department of Pediatrics, Endocrinology and Diabetology, Voivodship Children’s Hospital, Jana Karola Chodkiewicza 44, 85-667 Bydgoszcz, Poland; Faculty of Health Sciences, UJK, ul. Żeromskiego 5 25-369, Kielce, Poland

## Abstract

**Introduction:**

The adrenocortical rest tumours are the very rare entity in the pediatric population. They are usually found along the gonadal descent paths (celiac axis, the broad ligamen, the adnexa of the testes or the spermatic cord). They have been also described to occur at rare ectopic sites like intracranial locations, placenta, kidney, pancreas and liver.

**Clinical case:**

Here we present a unusual case of an ectopic, virilising, primary adrenocortical tumour localized in the spinal region in a 8 years-old-boy.

**Discussion:**

This is the first case of functional ectopic, adrenocortical tumour localized in the spinal region in a pediatric population. We discuss here the clinical presentation and the diagnostic challenges and provide the review of the literature.

## Case Report

A 7.5-year-old boy presented to the Department of Endocrinology with symptoms of virilisation (presence of pubic hair, enlargement of penis), acne and adult body odour.

Our patient was born after a full-term, unremarkable pregnancy and exhibited normal development until the age of 5 years. At the age of 5.5 years, the first symptoms of virilisation started to appear (pubic hair and enlargement of penis) as well as acceleration of growth velocity. On admission to the Endocrinology Department, on physical examination the patient was found to have pubic hair (Tanner stage III/IV), a large penis measuring around 6 cm. His height was 144 cm (+3.25SD) with a weight 28.1 kg (5–10 centile according to the height) , a growth spurt was diagnosed (growth velocity 9 cm/year), his bone age was two years advanced. There was acne limited to the region of his face and adult body odour but no axillary hair and no striae. Testicular size (2 ml each) and structure were normal for age. Moreover, neurologic examination revealed that the patient had difficulty in walking (he walked on his toes), increased leg reflexes and right site foot-clonus. His bowel and bladder function were normal as well his sensation.

Laboratory investigations revealed significantly raised serum androgen levels (Table 1), abnormally high concentrations of androgen metabolites in the urine steroid profile that were suggestive of a virilising tumour; and normal cortisol and ACTH levels.

**Table 1:** Laboratory findings prior to surgery and 9 days, 2 months and 7 months following surgery

	Androstenedione (N: 7.0-36) (ng/dl)	DHEA-S (N: 13–115) (ug/dl)	Testosterone total (N: 3–10) (ng/dl)	ACTH (N: 10–60) (pg/ml)	Cortisol (N: 5–20) (ug/gl)	LH (N: 0–0.3) (IU/I)	FSH (N: 0.2-3) (IU/I)
prior to surgery							
	**472**	**381.6**	**173.0**	**66.2**	13.0	2.07	2.56
following surgery							
9 days	**154**	36.1	13.4	37.6	12.2		
2 months	**111**	59.9	**31.3**	51.3	17.9	**3.15**	**4.6**
7 months	**110**	64	**51.7**	28.3	15.2	**46** ^a^	**12.5** ^a^

In bold are the values that are above the normal values

^a^The values following the LH-RH stimulation test

There was no androgen suppression observed during the 4-day Dexamethasone test (2 days of 2 mg/d, 2 days of 8 mg/d) but normal cortisol suppression after the small dose of Dexamethasone. Urine catecholamine and their metabolites levels were normal.

The abdominal and thoracic computed tomography (CT) scan and the adrenal magnetic resonance imaging (MRI) revealed no abnormalities. The ultrasound of the testes was normal. Adrenal scintigraphy (131 J-norcholesterol) showed no adrenal autonomic function.

**Fig. 1:**
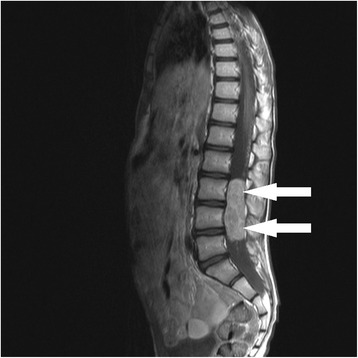
MRI of the spine. Sagittal contrast enhanced T1- weighted image shows a well-circumscribed, ovoid, strong enhancing intradural extramedullary mass at the L3-L4/5 level

**Fig. 2:**
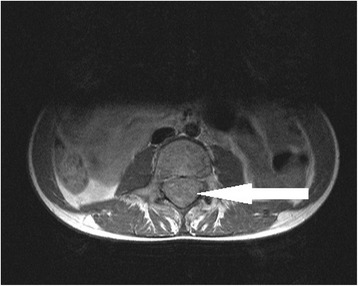
MRI of the spine. Axial T1-weighted post-contrast injection image: the mass occupies the entire cross-section of the spinal canal

The brain MRI was normal, however spinal MRI T2- and contrast enhanced T1-weighted images demonstrated an ovoid, well-circumscribed, intradural extramedullary enhancing mass measuring approximately 2.8x2.1x6.0 cm (Fig. 1). The mass occupied the entire cross-section of the spinal canal at levels L3 to L4/5 (Fig. 2). The presence of myxopapillary ependymoma was suspected.

The patient underwent an L3-L4 laminectomy. Upon opening the dura, an extramedullary mass was seen. There were calcifications within the arachnoid. The white-grayish mass measured 3.0x2.0x5.0 cm, appeared well-circumscribed, and showed no invasion of surrounding tissues. The lesion was resected totally.

**Fig. 3:**
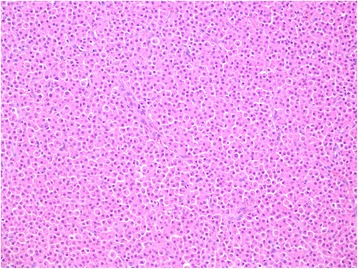
Histopathology of ectopic adrenocortical tumour in the spinal region. Hematoxylin and eosin stain, original magnification 200x. The tumour is comprised of round and polygonal cells with abundant eosinophilic cytoplasm

Microscopically features were of benign ectopic adrenocortical tumour (Fig. 3). On the IHCM (immunohistochemistry and microscopy) panel the cells were positive for melan-A, locally positive for synaptophisin and for CD56. The cells were negative for glial fibrillary acidic protein (GFAP), cytokeratins AE1/AE3 and chromogranin. The proliferative activity ki67 index was low (locally up to 3 %).

**Fig. 4:**
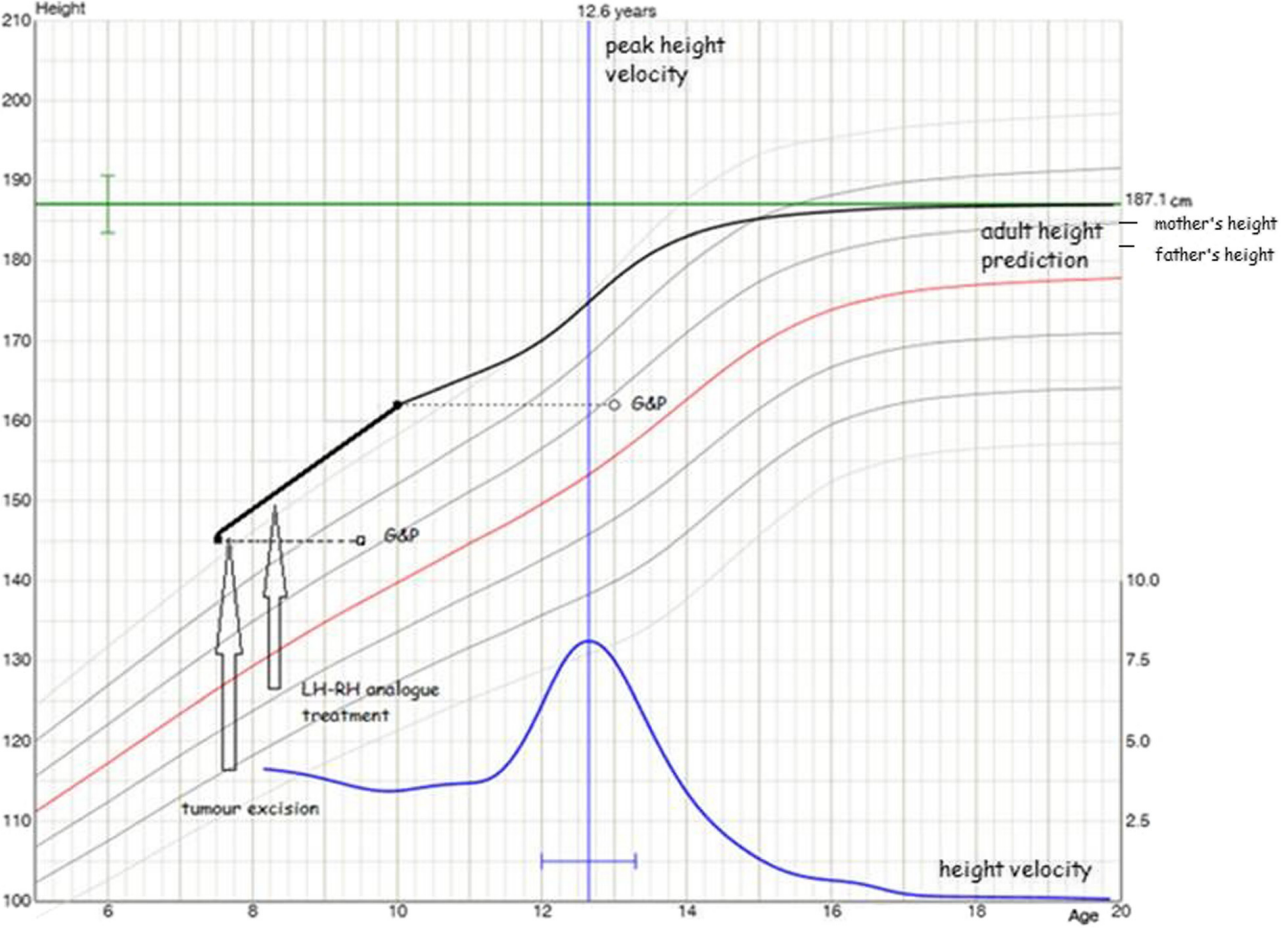
Growth chart of our patient. The figure shows his growth, his final height prediction that is based on his height, bone age and parental height. Also the curve of his height velocity and the age of predicted peak height velocity is indicated

Postoperative course was uneventful apart from transient urinary bladder function disturbances (difficulty urinating and patient required catheterisation regimen for several weeks). The patient was followed up and the normalization of androgen levels was observed in tests performed 9 days, 2 and 7 months after surgery (Table 1). Neurologic examination at 9 month post surgery was normal apart from lack of right Achilles tendon reflex. There were no abnormalities on control MRI of the brain and spine performed 9 months post surgery. Unfortunately secondary to the endocrine activity of the tumour, central precocious puberty occurred and patient started the treatment with long acting LH-RH analogue. The boy is now 10 years of age, his height is 162 cm and bone age is three years advanced. Fig. 4 shows his growth chart with final height prediction (the prediction was based on his height, bone age and parental heights) [1].

## Discussion

Adrenocortical tumours (ACT) are rare in children. It is estimated that there are 19–20 new cases of adrenocortical carcinoma in children and adolescents per year in the United States according to the Surveillance, Epidemiology and End Results Program (http://seer.cancer.gov/). There is a peak incidence before four years of age (0.4 cases per million) which then declines during the subsequent ten years. The incidence then rises to 0.2 per million during the late teens. The majority (60 %) of pediatric adrenocortical tumours occur in children less than five years [2]. There is a female predominance but only prior to adolescence (F/M 2:1 in young children, F/M 1:1 in adolescence) [3]. The most common presentation is virilisation, followed by hypercortisolism and hyperaldosteronism [4]. In adults there is predominance of non-hormone secreting tumours.

Adrenal rest tumours (ART) are collection of adrenal tissue that are found outside of the adrenal gland. The aberrant adrenal tissues are divided into heterotopia and the accessory adrenal gland. The former is derived from adrenal primordium that has migrated at an embryonic stage from neighboring organs such as kidneys or the liver; whereas the latter is the ectopia of fragmented adrenal tissues into the celiac axis, retroperitoneal cavity, uterus, broad ligament or testis [5].

Ectopic adrenocortical tumour in the region of the spinal cord is a very rare phenomenon [6,7]. It is very difficult to explain the pathogenesis of intradural spinal adrenal tissue. Karikari et al. speculated that premature separation of ectoderm from neuroectoderm before neurulation is completed may permit invasion of the neural groove by mesoderm tissue that is committed to the formation of adrenocortical tissue [8]. Whereas in cases with extramedullary adrenocortical tumours it has been suggested that ectopic rests of adrenal cells may gain access into the spinal canal via the sheath of an existing nerve or along the adventitia of an in-growing segmental lumbar artery from the aorta [6].

So far there have been only 6 cases of ARTs localized in the spinal region reported in the literature (Table 2).

**Table 2:** Intraspinal adrenal cortical adenomas reported in the literature

Ref. No.	Age (yr)/sex	Lesion location	Symptoms	Outcome
Kepes et al., 1990 [7]	8/F	Extramedullary, cauda eqina at L2	Bilateral leg pain behind the knees	Complete resection
Mitchell et al., 1993 [8]	16/F	Extramedullary adherent to L2 sensory nerve root	Pain in posterolateral right thigh	Complete resection
Mitchell et al., 1993 [8]	63/F	Extramedullary at cauda eqina	Lower back pain radiating to bilateral lower extremities	Complete resection
Cassarino et al., 2004 [10]	27/M	Intramedullary at conus medullaris	Lower extremity weakness, spastic paralysis, hypoesthesia below T10	Complete resection
Karikari et al., 2006 [9]	27/F	Intramedullary at conus medullaris with spinal dysraphism	Bilateral leg pain, urinary frequency	Subtotal resection, Residual, stable primary adrenal tumor
Rodriguez et al., 2009 [11]	5 mo/F	Extramedullary at T10-L2	Inability to bear weigth in lower extremity, irritability	Complete resection, recurrent tumor at 6 mo, chemotherapy

Those tumours occurred in the lower spinal region at a median patient age of 22 years (5 months to 63 years) and with a notable female predominance (5:1). In 2004 Cassarino reported the first case of an ACT within the CNS in a male patient [9]. This was also the first case of an intramedullary involvement. All of the tumours did not show atypia nor recurrence. In 2009 Rodriguez reported the first case of a tumour in a very young girl (5 months old) in whom although gross total resection was performed, 6 months later the lesion recurred [10].

It is difficult to separate benign from malignant tumours. There is no single criterion and histopathological classification may not be reliable. Recently several, molecular markers have been investigated as a diagnostic aid: overexpression of IGF-2, IGF-1R, somatic p53 mutations, ki67 index and loss of heterozygosity of 11q13 [11–14]. Morphological signs of tumour carcinogenic potential is tumour weigh (>100 g) and diameter (>5 cm)[15].

All reported, up till now, tumours in the spinal region were non functional. In the literature there were only few cases of clinically-functioning adrenal rest tumours [16] but all of them were localized outside the central nervous system. None of the cases reported so far noted signs of clinical virilisation/feminisation, Cushingoid features or abnormal serum cortical levels.

In the present study, the young boy exhibited typical symptoms of virilizing tumour with high serum levels of androgens and characteristic urine steroid profile, normal cortisol circadian rhythm and normal suppressed serum cortisol level following Dexametasone administration.

His clinical signs were pubic hair, enlargement of penis, growth acceleration, adult body odour, acne. We encountered difficulty in localization the tumour. Neither ultrasonography, CT or scintigraphy did not reveal the tumour and as well as brain MRI were all normal. Definite diagnosis was made with MRI of the spine. The diagnosis was of a benign adrenal tumour. Vascular invasion and metastasis were not found.

Considering the final, adult height prediction in children with advance bone age due to hypersecretion of androgens, it is reported in the literature, that early recognition and treatment can normalize the growth pattern [17]. Although in our case the central precocious puberty has led to further advancement of the bone age, the boy’s predicted height based on his actual height, advanced bone age and parental heights does not differ much from the target height.

In summary we report the unique occurrence of a primary adrenocortical tumour in the spinal region of a young boy. To the best of our knowledge this is the first report of a ACT with the steroidogenic capacity within that localization. Although the occurrence of adrenocortical tumours in central nervous system is rare, pathologist should be aware that ectopic tumours both benign and malignant that may arise at this localization. Even in cases with no frank histological malignancy features present the patient should be followed-up.

## Consent

Written informed consent was obtained from the patient’s parents/legal guardians for publication of this case report and any accompanying images. A copy of the written consent is available for the review by the Editor-in-Chief of this journal.
